# Clarification of Governance Relevant to the Sustainable Management of Marine Species and Habitats within the United Kingdom: An Overview of Regional, National and International Authorities, Advisories, Legislation and Designation Types with Summary Schematic Tool

**DOI:** 10.1007/s00267-018-1064-z

**Published:** 2018-06-17

**Authors:** C L Mackenzie, J Vad, R MacPherson

**Affiliations:** 1 School of Energy, Geoscience, Infrastructure and Society, Heriot-Watt University, Riccarton, Edinburgh, EH14 4AS UK; 2 School of Geoscience, Grant Institute, University of Edinburgh, Edinburgh, EH9 3FE UK

**Keywords:** Marine conservation, Marine management, Marine governance, Priority marine feature, Priority marine habitat, United Kingdom

## Abstract

Marine management developments are occurring across the United Kingdom with the major aim to ensure economic growth and security of marine resources via the provision of legislative guidelines for sustainable management of activities within the marine environment. Many of these directives also provide guidance for maintaining ecologically valuable and/or endangered species and habitats that exist alongside, and may also support, marine activities/use. Marine governance is largely guided by several key directives laid out and implemented by governing authorities of Europe, the United Kingdom and those countries comprising the United Kingdom, and in line with several international conventions. The directives set out by each authority or convention may act discretely but more often tend to overlap, which can lead to confusion about the relevant marine conservation requirements and objectives that must be fulfilled for a given region, site or feature. Additionally, management objectives driven by the same legislation may oppose one another, adding further complexity to the matter. This article aims to provide an overview of governance that holds relevance to managing marine habitats and species, especially those deemed sensitive, ecologically valuable and/or endangered. A general overview and summary schematic tool of the marine governance, legislation and designations within each level of authority for the United Kingdom are provided. Additional consideration of the implications for legislation upon the United Kingdom leaving the EU is briefly discussed and a comparative case study of two marine habitats of high conservation value is provided to demonstrate how different sites/features may have considerably different management requirements.

## Introduction

Currently, a number of marine management developments are taking place across the United Kingdom (UK) and Europe with the primary aim to ensure the future sustainability of marine resources. Predominant marine governance comes from the European Commission (EC) via a number of key directives but government mandates at the regional and national scale also play essential roles. Consequently, regional and national governments from across the UK are working to develop, improve and implement their own marine strategies in order to meet the requirements of such EC directives as well as those that may be set out in their own legislations. However, the considerable level of uncertainty regarding how marine management objectives and requirements may change under the UK leaving Europe (i.e. Brexit) adds a degree of complexity to the future direction of marine management and thus poses an additional challenge to managers and policy makers. At the same time, planning frameworks at regional and national scales, consisting largely of integrated coastal zone management (ICZM) and marine spatial planning (MSP) are also emerging.

Currently, the UK remains a member state of the European Union (EU) and comprises four countries: England, Wales, Scotland and Northern Ireland. While the UK government acts as the responsible authority for aspects such as the constitution, international relations and defence, national security, nationality and immigration, various other powers including agriculture, forestry, fisheries, the environment and planning have been devolved to the national governments in Wales (Welsh Government), Scotland (The Scottish Government) and Northern Ireland (Northern Ireland Executive). Consequently, when working towards effective marine management within a specific region or examining a species with distribution falling across multiple regions, one must consider governance, legislation and designations at multiple administrative levels including those of EU, UK and national governments. Additionally, there are a number of international conventions that must be considered.

The predominant objective of marine governance legislation across administrative levels is to ensure sustainable economic sustainability and security of marine resources. Legislation provides a set of rules that ensures any marine operations and/or activities contribute to this aim. Likewise, directives promote the development of strategies at regional, national and international scales in order to address common challenges and to develop economic growth (i.e. blue growth) across natural resources, energy, trade and security sectors. Government directives at each level also contribute to marine research objectives via provision of research funds, improved collation and accessibility of marine data and enhanced communication (e.g. forums) across regions and disciplines.

However, while marine management directives, objectives and obligations set out by each level of administration or convention can act discretely, they more often overlap. This can lead to confusion or uncertainty around the legislative guidance for a given region, site or feature. This is particularly noted for parties such as marine researchers, businesses, developers, consultants and nongovernmental organisations that, while working within marine environments and thus directly involved in management, are often removed from policy development and implementation. Consequently, they may be either unacquainted with or unclear of the current collective and overlapping requirements set out by the various government departments and their advisory bodies.

Many of the driving legislative texts behind sustainable use of marine environments also provide guidance for maintaining ecologically valuable, sensitive and/or endangered species and habitats (e.g. management of vulnerable/endangered species, implementation of marine-protected areas (MPAs) etc.), which is the focus of this article. While the maintenance of such species/habitats can be key for economic sustainability (e.g. maintaining fish-breeding grounds for safeguarding fisheries stocks), and thus economic and environmental objectives may be compatible, an inherent conflict often exists in resource management between meeting objectives for marine activities concentrated on sustainable resource use and development whilst maintaining valuable species and habitats in areas where these activities are focussed. This conflict is illustrated by various legislative directives where mandated requirements for resource conservation and resource development come up against each other. For example, the extraction of living resources through fishing activities and the production of living resources from aquaculture directly and indirectly impact coastal and deep marine environments (Halpern et al. 2007; JNCC 2016). Likewise, the extraction of nonliving resources such as hydrocarbons and the generation of renewable energies require the installation of heavy man-made infrastructures in the marine environment (Halpern et al. 2007; JNCC 2016), potentially impacting marine environments and/or disturbing processes (e.g. hydrodynamics) that are relied upon by ecosystems of such environments. Additionally, shipping activities continue to increase and waste disposal from shore-based human activities brings impacts to offshore environments (Halpern et al. 2007; JNCC 2016). Finally, global climate change (including ocean warming and acidification, increased incidence of hypoxic events and changes in salinity) exerts further pressure on these environments. In light of these collective and mounting pressures, effective marine governance, including a clear understanding of relevant authorities, directives and designations, is paramount.

Clarification of key marine governance that is relevant to marine conservation objectives for the sustainable management of marine species and habitats is the focus of this paper. Conservation is defined here according to WRI IUCN and UNEP (1992); in brief, it refers to the sustainable use of resources to ensure that the environment may provide sustainable economic benefit to current generations whilst maintaining its potential for future generations. Accordingly, this includes the maintenance of marine habitats/species that exist alongside marine activities/development, in order to safeguard marine resources for sustainable use and minimise impact, particularly on species/habitats deemed ecologically valuable, sensitive or in decline/endangered. As stated previously, many of the guiding directives and resulting objectives which inform marine conservation purposes are driven by goals for sustainable economic use of marine environments. Consequently, it is important to consider both use and preservation objectives alongside one another and as equivalent entities within the framework for sustainable management of marine resources.

To our knowledge, there is currently no unified resource that provides a clear summary of marine governance at UK, European and international levels (including governing bodies, advisors, legislation etc.). The current work provides a summary of key legislation and designation types that should be considered when working towards objectives for the sustainable management of marine habitats and species within the UK. This will aid in clarification of governance for those working within marine environmental sectors and consequently contribute to a more effective MSP process and increasingly streamlined marine management. Additionally, a summary schematic is provided in order to highlight key information at national and local scales, thus serving as a simple yet effective reference tool. Finally, a comparison of an offshore site and coastal site for two marine habitats of high conservation value is provided to demonstrate how different sites/features may have considerably different governance requirements.

## United Kingdom

### UK-wide

The Department for Environment, Food and Rural Affairs (DEFRA) is the UK government department responsible for management of UK seas. The Joint Nature Conservation Committee (JNCC) advises both DEFRA and the UK’s four devolved administrations on UK-wide and international marine nature conservation. The driving legislation for UK marine matters is the Marine and Coastal Access Act 2009 (also known as the UK Marine Act), which sets out the terms for creation of Marine Conservation Zones (MCZs). The purpose of MCZs is to protect nationally important marine wildlife, habitats, geology and geomorphology in English and Welsh territorial waters and UK offshore waters but does not extend to Scotland. While MCZs are also present in Northern Ireland, governance is established by Northern Ireland legislation. The Marine and Coastal Access Act also works to meet international marine initiatives including the EU Marine Strategy Framework Directive (MSFD) and EU Maritime Spatial Planning Directive (JNCC 2016).

JNCC is responsible for identifying Special Areas of Conservation (SACs) in the UK offshore marine area beyond 12 nm within British fishery limits and the seabed within the UK Continental Shelf Designated Area (JNCC 2016). SACs are protected sites designated under the EC Habitats Directive (Directive 92/43/EEC) for protection of species and habitats listed under Annexes I and II of the directive. JNCC can also recommend SACs for habitat features associated with the seabed only in areas that are within the UK Continental Shelf Designated Area but beyond the exclusive economic zone. The Offshore Marine Conservation (Natural Habitats &c.) Regulations 2007 fulfils the requirement of European law (Habitats and Birds Directives) beyond inshore waters.

JNCC also works with statutory conservation agencies in Scotland (Scottish Natural Heritage), England (Natural England), Wales (Natural Resources Wales) and Northern Ireland (Department of Agriculture, Environment and Rural Affairs) to identify sites for designation as marine Special Protected Areas (SPAs). SPAs are protected sites designated under Article 4 of the EC Birds Directive (79/409/EEC) (JNCC 2016). Similarly, JNCC provides guidelines for the production and revision of guidelines for selection of Sites of Special Scientific Interest (SSSIs). Accordingly, the statutory conservation agencies of Great Britain (i.e. England, Wales and Scotland) have a duty under the UK Wildlife and Countryside Act 1981 to notify of any site which is deemed to be ‘of special interest by reason of any of its flora, fauna or geological or physiographical features’ (JNCC 2016).

Additional marine conservation guidance for the UK comes from the UK Post-2010 Biodiversity Framework (succeeding the UK Biodiversity Action Plan), which covers the period 2011–2020 and was developed to meet requirements of international biodiversity conventions: the Convention on Biological Diversity’s (CBD’s) Strategic Plan for Biodiversity 2011–2020 and the EU Biodiversity Strategy (EUBS) (JNCC 2013). Details of the Post-2010 Biodiversity Framework can be found in the Implementation Plan report published by JNCC in November 2013 (JNCC 2013).

### England

While some governance is devolved to local authorities in England, England does not have a national devolved government, and consequently marine management and planning remain under the duty of the wider UK government (i.e. DEFRA). However, DEFRA does sponsor the Marine Management Organisation (MMO) and Natural England, both executive non-departmental public bodies, which are responsible for licencing, regulating and planning marine activities in the territorial seas around England and Wales, and advising the government on the natural environment in England, respectively. Marine activities and conservation designations (e.g. of MCZs) in England fall under the legislation of the Marine and Coastal Act (2009).

### Northern Ireland

The Government of Northern Ireland’s Department of Agriculture, Environment and Rural Affairs (DAERA) has the primary responsibility for managing marine environments of Northern Ireland. DAERA’s stated responsibility is to protect Northern Ireland’s coastal and marine environment via legislation, licencing and permits and conservation activities (DAERA 2017). The Council for Nature Conservation and the Countryside is the statutory advisor to DAERA, advising on any matters related to marine conservation, as well as the establishment and management of Marine Nature Reserves (MNRs) (DAERA 2017). The driving legislation for marine activities in Northern Ireland is the Marine Act (Northern Ireland) 2013, which applies to the Northern Ireland inshore region (covering Northern Irish territorial water, 12-nm limit) and includes all tidal rivers and sea loughs. The Marine Act (Northern Ireland) 2013 covers all aspects of marine planning (e.g. preparation of marine plans for the inshore regions), nature conservation (e.g. designation of MCZs) and marine licencing. Northern Ireland has several types of MNRs including Areas of Special Scientific Interest (ASSIs), MCZs and MNRs. ASSIs are the equivalent of SSSIs (in Wales, England and Scotland) and are set up to protect Northern Ireland’s plants, wildlife and geological features, and while sites are predominantly terrestrial, a number fall within marine environments. MCZs are areas designated to protect species, habitats and geological features of national importance under the Marine Act (Northern Ireland) 2013, and will be part of the wider UK network of protected sites while MNRs are sites designated under the Nature Conservation and Amenity Lands (Northern Ireland) Order 1985 (DAERA 2017).

### Scotland

Marine Scotland is a directorate of the Scottish Government and is responsible for the integrated management of Scotland’s seas. The principal advising body to Marine Scotland is Scottish Natural Heritage (SNH) that works to secure the conservation and enhancement of nature and landscape and promote sustainable use and management of Scotland’s natural environment, working with various other stakeholder groups (SNH 2017). The key legislative driver regarding Scotland’s marine environment is the Marine (Scotland) Act (2010), which covers international, national and regional aspects of marine management and spatial planning, including a strategic marine-planning system, a streamlined marine-licencing system and improved marine nature conservation measures, improved measures for the protection of seals and improved enforcement measures (JNCC 2013). Internationally, the Marine (Scotland) Act (2010) is in accordance with the EU Directive 2014/89/EU (Maritime Spatial Planning) and the EU MSFD. The Marine (Scotland) Act (2010) also creates Scotland’s first National Marine Plan (NMP), which covers the management of inshore waters (out to 12 nm) and offshore water (12–200 nm), as well as 11 Scottish marine regions (covering Scottish territorial water, 12-nm limit) that will be managed by Marine Planning Partnerships according to Regional Marine Plans (RMPs) that meet the requirements of the NMP. Legislative requirements for RMPs as set out by the NMP include assessing the condition of each region, summarising the significant pressures and impacts of human activity and stating the contribution of MPAs and other designated areas to the protection and enhancement of the region. The Marine (Scotland) Act (alongside the UK Marine and Coastal Access Act) also provides powers to Scottish ministers to designate Nature Conservation Marine Protected Areas (NCMPAs) (SNH 2017). SNH and Marine Scotland have also worked with JNCC (UK-based) to develop a classification of Priority Marine Features (PMFs), which refers to habitats and species of marine nature conservation priority in Scottish waters.

### Wales

In Wales, the management of the marine environment is the responsibility of the Welsh Government. Natural Resources Wales is the principal advisor on marine affairs to the Welsh Government, and additionally advises industry and the wider public about issues relating to the marine environment. There is no specific Welsh marine legislation as in Scotland and Northern Ireland. Previously, MNRs were established under the Wildlife and Countryside Act 1981 for England and Wales but the introduction of the UK Marine and Coastal Act (2009) has now replaced MNRs with MCZs.

**Fig. 1:**
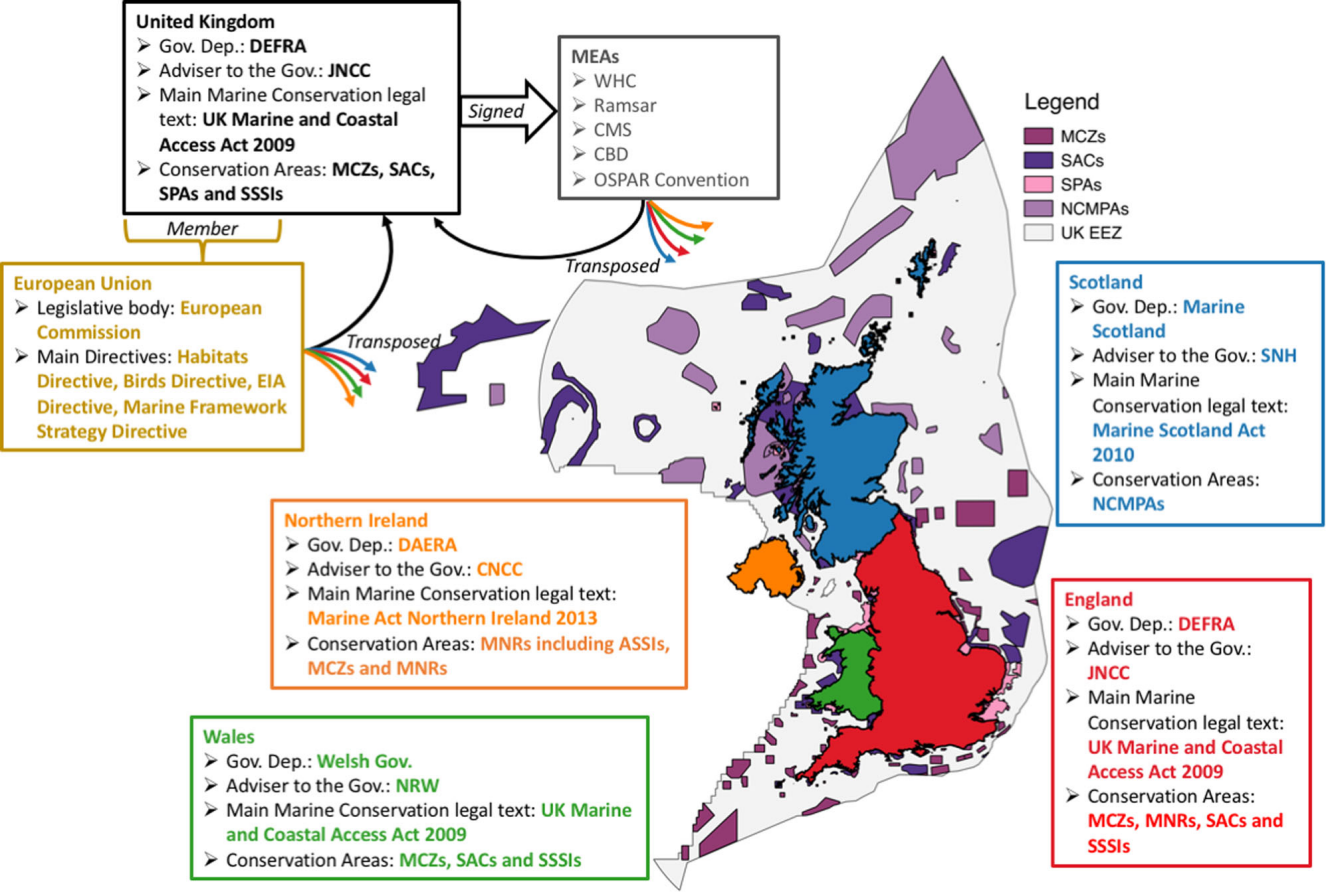
Summary schematic of governance relevant to the sustainable management of marine species and habitats within the UK. The main authorities, advisories, legal texts and designations for the UK, England, Northern Ireland, Wales and Scotland, and at European and international levels are included

Table 1 provides a summary of the main UK marine legislation (covering that which is specific to England, Scotland, Northern Ireland and Wales, and across the UK as a whole) relevant to the sustainable management of marine species and habitats within the UK, including a list of key objectives and designations. Figure 1 acts as a summary schematic of guiding legislation, authorities, advisories and designations, and highlights how marine governance varies across the UK region.

**Table 1:** Overview of governance relevant to the sustainable management of marine species and habitats within the UK including major legislative texts/conventions and corresponding key objectives and designations

	Text	Area	Date	Key objectives	Marine conservation designations
UK legislation	Marine and Coastal Act 2009	England and Wales	2009	Creation of MMO	MCZ
				Creation of a strategic marine-planning system	
				Improvement to the marine-licencing system	
				Designations of MCZ	
				Improvement to fisheries (marine and freshwater) management	
				Improvement to coastal access	
				Improvement to coastal and estuarine management	
	Marine (Scotland) Act (2010)	Scotland	2010	Creation of a strategic marine-planning system	MPA
				Improvement to the marine-licencing system	
				Improvement to marine nature conservation measures with designations of MPAs	
				Improvement to seals protection measures	
				Improvement to enforcement measures	
	Marine Act (Northern Ireland) 2013	Northern Ireland	2013	Improvement to marine planning	MCZ
				Improvement to marine nature conservation measures with designations of MCZs	
				Improvement to the marine-licencing system	
European directives	EC Birds Directive (79/409/EEC)	EU	1979	Maintenance of populations of wild bird species	SPA
				Identification and classification of SPAs	
				Establishment of general protection scheme for all wild birds	
				Restriction on sales and keeping of wild birds and on hunting and falconry	
				Prohibition of large-scale nonselective means of bird killing	
				Encouragement of relevant research	
				Requirements to introduction of nonnative birds to protect local biodiversity	
	EC Habitats Directive (92/43/EEC)	EU	1992	Maintenance or restoration of protected habitats and species	SAC
				Contribution to a coherent European network of protected sites by designation of SACs	
				Improvement to conservation measures to appropriately manage SACs	
				Undertaking surveillance of habitats and species	
				Strict protection of species listed in Annex IV of the directive	
				Reporting on implementation every 6 years	
	EU Water Framework Directive (2000/60/EC)	EU	2000	Establishment of a framework for the protection of inland surface waters, estuaries, coastal waters and groundwater	
				Requirements for all aquatic ecosystems to meet ‘good status’ before 2015	
				Establishment of river basin districts with relevant river basin management plan	
				Reporting on implementation every 6 years	
	EU Marine Strategy Framework Directive (2008/56/EC)	EU	2008	Establishment of a legislative framework for an ecosystem-based approach to the management of human activities which supports the sustainable use of marine goods and services	
				Requirements for marine environment to reach ‘Good Environmental Status’ by 2020	
				Establishment of four European marine regions required to develop their own marine strategic plans	
	EU Maritime Spatial Planning Directive (2014/89/EU)	EU	2014	Managing human activities in an efficient, safe and sustainable way	
				Reducing conflicts between sectors and activities	
				Increasing cross-border cooperation to develop shared infrastructure but also a coherent network of protected areas	
				Protection of the environment through early identification of impact and opportunities for multiple use of space	
International conventions	Ramsar Convention	Where ratified	1976	Designation of wetlands of international importance as Ramsar sites	Ramsar sites
				Promotion of the wise use of all wetlands in the territory of each country	
				International cooperation to further the wise use of wetlands and their resources	
	World Heritage Convention	Where ratified	1984	Designation of sites of international cultural and/or natural importance as World Heritage Sites within each state party	World Heritage Sites
				Requirement of each state party to protect World Heritage sites	
				Creation of the World Heritage List	
				International cooperation to further the wise use of wetlands and their resources	
	Convention on the Conservation of Migratory Species of Wild Animals	Where ratified	1985	Conservation of migratory species and their habitats by providing strict protection for endangered migratory species	
				Concluding multilateral agreements for the conservation and management of migratory species	
				Encouraging cooperative research activities	
	Convention on Biological Diversity	Where ratified	1994	Conservation of biological diversity	
				Sustainable use of biological diversity	
				Fair and equitable sharing of the benefits arising from the use of genetic resources	
	OSPAR convention	Where ratified	1992	Providing a comprehensive approach to address all sources of pollution affecting the maritime area	
				Adoption of the Biodiversity Strategy requiring the identification of ecological quality objectives for the North Sea, the development of lists of species and habitats in need of protection, identification and selection of marine-protected areas and the prevention and control of adverse impacts from human activities

## European Union

The EU has a comprehensive environmental policy framework in place and has been a key driving force in the establishment and implementation of nature conservation in the UK and other member states (Boyes and Elliott 2014). The EC is the governing body of the EU and EC directives bind specific member states to a given target, with each member state and respective national authorities in control of the form and methods used to meet said targets. All EC directives have been transposed into law in the respective UK territories. Under the Scotland Act (1998), for example, the Scottish Government is responsible for creating and passing laws, which will help implement EU law and put the legislation into place. Similarly, in England, Wales and Northern Ireland, transposed legislation helps implement all EU law and some international environmental targets.

### Habitats Directive and Birds Directive

The Habitats Directive (92/43/EEC) and the Birds Directive (2009/147/EC) are the cornerstone of European nature conservation. The nature directives call for development of conservation management policy and indicators of condition in MPAs (Council Directive 92/43/EEC, 1992). Stemming from both directives is an EU-wide network of protected areas; Special Protected Areas (SPAs) for birds and Special Areas of Conservation (SACs) collectively known as the Natura 2000 network. In the marine environment, the Natura 2000 network consists of more than 3000 SACs and SPAs and covers more than 5% of the total EU marine area (>300,000 km^2^) (as of June 2016) (EC 2016). The Habitat Directive has two main threads; a list of species under strict protection (European protected species, EPS) and the Natura 2000 network, which offers site protection. ‘Favourable conservation status’ of these sites and species is required nationally and across regions under EU jurisdiction. Each nation is responsible for the maintenance and periodic reporting of the conservation status of annexed species and habitats and any negative impacts. Member states are subject to penalties for non-compliance and degradation of annexed species and habitats by the EU (EEA 2015).

### Marine Strategy Framework Directive

The Integrated Marine Policy (IMP) for the EU aims to develop trans-border marine management framework, encouraging ‘Blue Growth’ through holistic MSP and management (EU 2007; EU 2008). One of the main EU directives generated from the IMP is the Marine Strategy Framework Directive 2008/56/EC (MSFD) which calls for 'good environmental status' (GES) across marine areas in all member states by 2020. It is the first EU legislative instrument related to the protection of marine biodiversity, with the explicit objective that 'biodiversity is maintained by 2020'. In order to achieve this status, priority habitats (i.e. defined as ‘threatened and/or declining species and habitats’ under the OSPAR Convention for the Protection of the Marine Environment of the north-east Atlantic 1992) will need to be assessed, maintained and managed for both current conditions, as well as future conditions under climate change scenarios (MSFD 2008). In order to achieve GES by 2020, each EU member state is required to develop a strategy for its marine waters. This 'Marine Strategy' must be kept up-to-date and reviewed every six years to comply with the directive’s adaptive management approach. The directive legislates an ecosystem approach to marine management encouraging protection alongside sustainable use. In the UK, the MSFD was transposed through the Marine Strategy Regulations 2010. Implementation of the programme of measures is delegated to each national government for features present in England, Northern Ireland, Scotland and Wales. The MSFD is broken down into 11 key quality descriptors; three biological and eight pressure-related with each of these high-level descriptors broken down into a set of criteria and furthermore into indicators. GES will be assessed according to the indicators developed for all criteria of the relevant high-level descriptors relating to the OSPAR priority habitats (HM Government 2012). The MSFD applies to all coastal and offshore marine areas up to 200 nm (excluding transitional waters like estuaries, as defined by the Water Framework Directive 2000/60/EC).

### Water Framework Directive

The Water Framework Directive (WFD) (2000/60/EC) seeks to improve water quality in inland, surface, ground, transitional and coastal water bodies. The directive works on a 6-year cycle, with the last target date in 2015. There are a range of assessment elements, much like the MSFD, but are focussed on the biological and chemical status of all water bodies. Water bodies are classified on an ecological and chemical scale aiming for ‘good ecological status’ and ‘good chemical status’ for all water bodies. In terms of the marine environment, the WFD is applicable to transitional waters (defined as estuaries, sea lochs, and coastal lagoons) and coastal waters to 1 nm (3 nm in Scotland). For chemical status, this boundary stretches to territorial waters, 12 nm. There is considerable overlap between the WFD and the MSFD in coastal waters. Within the MSFD, there is an explicit recognition that the MSFD applies in coastal water only where GES is not covered by the WFD like noise, litter and some aspects of biodiversity (zooplankton, seabirds, coastal water fish and marine mammals) (HM Government 2012). As with other directives, they have been transposed into national law. The implementation of the legislation is carried out by the Scottish Environmental Protection Agency and Marine Scotland in Scotland, the Northern Ireland Environmental Agency in Northern Ireland, the Environmental Agency and DEFRA in England and Natural Resources Wales in Wales.

### Maritime Spatial Planning Directive

The EC Maritime Spatial Planning (MSP) Directive 2014/89/EU is another key legislation stemming from the IMP. The MSP Directive requires all states to design and implement marine spatial plans, giving them the freedom to do this at national, regional or local scales. As with other EU policies, there are minimum common targets and timescales; plans need to be in place by Spring 2021, they should include all human activities with land–sea interactions, public and stakeholders must be involved throughout the planning process and the most effective future spatial development of the marine area must be identified (EU 2011a). Activities considered within the MSP include fishing, aquaculture, renewable energy, offshore oil and gas activities and maritime transportation (EU 2011a). The MSP Directive seeks to encourage ‘Blue Growth’, a key EU initiative for sustainability and holistic management of the maritime area (Flaguel 2010). National legislation like the Marine Scotland Act (2010) included MSP measures. The MSP Directive ensures that all members states contribute to marine management on equal terms.

### EU Biodiversity Strategy

To complement binding legislation, the EU adopted the EU Biodiversity Strategy in 2011 following the commitments made at the 10^th^ Convention of Biological Diversity (CBD) in 2010. The vision set out by the EU was to protect, value and restore biodiversity, natural capital and ecosystem services (EU 2011b). This was partly adopted in recognition of the failure of the EU to meet 2010 biodiversity targets set by the EU in 2001. The EU biodiversity strategy contains six targets: full implementation of EU nature legislation to protect biodiversity, better protection for ecosystems and more use of green infrastructure, more sustainable agriculture and forestry, better management of fish stocks, tighter controls of invasive alien species, and bigger EU contribution to averting global biodiversity loss. The strategy also includes a commitment to help halt global biodiversity loss based on the CBD. As of 2015, only one of the six targets was on track (others progress classified as not significant or insufficient), invasive species target five, according to the mid-term report (EC 2016). The 2020 headline target showed no significant progress and overall, biodiversity loss and degradation of ecosystem services in the EU has continued since the 2010 baseline (EEA 2016). This strategy links with the nature directives, which have no set deadlines. The EU is aiming for all 100% of habitats in favourable conservation status by 2020.

Table 1 provides a summary of the main EU marine legislation relevant to the sustainable management of marine species and habitats within the UK including a list of key objectives. Please refer to Fig. 1 for a summary schematic.

### Considering the role of Brexit

In June 2016, the UK voted to leave the EU, commonly referred to as 'Brexit'. The EU has been instrumental in driving nature conservation policy across European seas, fixing ambitious targets through directives that have required all countries to take action for marine conservation and planning. Further, many of the most important UK environmental policies and priorities are those that have emerged via the EU (Boyes and Elliott 2014). Consequently, any scenario in which the UK withdraws from the EU could have significant implications for the marine environment (Boyes and Elliott 2016).

While the political, economic, social and environmental impacts of Brexit are largely unknown, the application of EU policy under which the UK is currently bound would likely change under different Brexit scenarios and consequently result in varying degrees of policy implications (IEEP 2016). Ultimately, the final outcomes will depend on negotiations with the EU; however, if the UK were to remain part of the European Economic Area (EEA), it is assumed that most environmental law would still apply (Burns et al. 2016). There would, however, be some major exceptions; most notably the Habitats and Birds Directives. Although these directives have been transposed into UK law, the UK would have the freedom to loosen or change all aspects of the legislation and be free of EU pressures to achieve favourable conservation status. The UK would still be bound to the ambitious targets of the MSFD, WFD and most other environmental policies, but would be unable to influence their development or negotiate future target setting and still be subject to penalties for noncompliance. If the UK were to completely break from the EU, no EU legislation would apply. The directives which have been transposed into law in the respective UK nations would still apply but the governments would have the freedom to change these laws over time to suit their own requirements (Burns et al. 2016). Additionally, the UK would still be required to meet the standards of many directives in order to trade with the EU, which would need to be negotiated throughout the Brexit process. While there is a risk that the UK could loosen environmental legislation in order to increase UK competitiveness, the EU has developed blanket policies which prevent states from increasing competitiveness by reducing environmental standards (e.g. increasing fishing quotas). According to the Institute for European Environmental Policy (IEEP), the UK would likely seek agreements on a voluntary basis with Europe in relation to the marine environment and fisheries (IEEP 2016). For a more in-depth examination, Boyes and Elliott (2016) provide a comprehensive overview of how current EU legislation that contributes to the protection and management of UK marine environments may change under varying Brexit exit scenarios, and what the implications of such changes could be for UK marine governance.

## International Conventions

Since the 1970s, the UK has been a contracting party to many Multilateral Environmental Agreements (MEAs), often introduced and developed by the United Nations (UN) (Kelemen and Knievel 2015). These MEAs share a common organisation. Overall, they have signatories (states not legally bound to the agreement) and parties (states legally bound to the agreement after its entry into force). The Conference of the Parties (COP) to the agreement constitutes its final decision-making body and can, for example, set up subsidiary bodies, agree on new obligations to parties and review the implementation of current obligations. The subsidiary bodies are present to prepare COP decisions, while a secretariat to the agreement is often set up to manage meetings and communicate decisions. The secretariat often works within a UN programme or other international organisations (Oberthür 2002).

An overview of five major MEAs of which the UK is a party and which have specific importance for marine conservation is provided. The Convention on International Trade in Endangered Species of Wild Fauna and Flora also constitutes a major convention on conservation issues but as its primary focus is the trading of wildlife, it has less relevance to the current topic and thus will not be discussed. Additionally, although the UN Framework Convention on Climate Change is of great significance and relevance to global and local conservation issues, it will not be considered here as it does not directly cover topics of marine conservation.

### Convention on Wetlands of International Importance especially as Waterfowl Habitat (Ramsar)

The Convention on Wetlands of International Importance especially as Waterfowl Habitat or Ramsar Convention aims at conserving and developing 'wise use' of wetlands via the listing of Ramsar sites (Ramsar 2016). The convention maintains a wide definition of wetlands in order to include as many sites as possible and strives to meet the following goals: (1) to designate wetlands of international importance; (2) to promote the wise use of all wetlands in the territory of each country, and (3) to establish international cooperation to further the wise use of wetlands. A national Ramsar Committee acts in an advisory capacity to assist governments in the implementation of the convention. These committees have a 3-year plan in accordance with the Convention’s Strategic Plan. The current 4^th^ strategic plan of the convention set forward 14 priority focus areas, achievable by 2024 (Ramsar 2016).

### World Heritage Convention

The World Heritage Convention (WHC) or Convention Concerning the Protection of the World Cultural and Natural Heritage, seeks to identify and conserve sites that are of significant cultural and/or natural universal value. World Heritage Sites (WHSs) are therefore preserved for all humanity and the WHC ensures their protection through international cooperation. Parties to the convention are required to list sites within their territories and take appropriate measures to protect them (Abdulla et al. 2014). Sites must fulfil at least one of ten criteria given by the Operational Guidelines for the Implementation of the WHC. Of the ten criteria, six are directly relevant to conservation issues (UNESCO 2015). Furthermore, 46 of the 981 WHSs are currently enlisted for their important marine values. Parties are encouraged to identify and nominate more marine sites of universal value to the WHC Global Strategy for a representative and balanced World Heritage List (UNESCO 2015). In the UK, the Department for Culture, Media and Sport is responsible for the country’s general compliance with the WHC and for nominating sites in England. Administrations in Wales, Scotland and Northern Ireland are responsible for choosing sites for nomination from their areas.

### Convention on the Conservation of Migratory Species of Wild Animals (CMS)

The CMS was adopted to help converse terrestrial, marine and avian migratory species. The CMS includes two appendices. Appendix I lists migratory species that are endangered and where urgent international cooperation is needed, while Appendix II lists other species that would benefit from international agreement under the convention. In general, the CMS promotes cooperative research, the adoption of protection measures and the conclusion of multilateral agreements. In this sense, the CMS acts as a framework convention under which specific agreements (with their action plans) can be discussed.

The UK has currently ratified four legally binding agreements under the convention (JNCC 2016). Three of these agreements are relevant to marine conservation (CMS 2017), including the African-Eurasian Migratory Waterbird Agreement, the Agreement on the Conservation of Small Cetaceans in the Baltic, North East Atlantic, Irish and North Seas and the Agreement on the Conservation of Albatrosses and Petrels. In addition to these agreements, the UK has also ratified a series of Memoranda of Understanding (MOU), such as MOUs on Migratory Sharks and Pacific Islands Cetaceans. MOUs are not legally binding but establish an official partnership for engagement in appropriate conservation actions (CMS 2017).

### Convention on Biological Diversity

The Convention on Biological Diversity (CBD) was created by the UN to solve critical issues regarding the environment and is the first global agreement on the topic of environmental conservation. It is regarded as the most significant product of the 1992 UN Conference on the Environment and the Development (CBD 2016). The CBD’s main goal is to ensure (1) the conservation of biodiversity; (2) the sustainable use of the components of biodiversity and (3) the fair sharing of benefits arising from the sustainable use of the environment. To achieve these objectives, the CBD develops targets laid out in Strategic Plans for Biodiversity (each plan lasting for about a decade), but leaves the responsibility of accomplishing these targets on the parties. The countries that ratified the convention are therefore required to develop national and/or local biodiversity strategies and action plans (CBD 2010). The current Strategic Plan for Biodiversity of the CBD was adopted during the COP-10 in Nagoya in 2010 and sets out targets to be achieved by 2020. These targets are called the Aichi targets and are considered as a framework enabling coherent plans and actions to be taken between the parties. The 20 targets are divided into five strategic goals. The main mission of the current Strategic Plan for Biodiversity as set out by the CBD is to 'take effective and urgent action to halt the loss of biodiversity in order to ensure that by 2020 ecosystems are resilient and continue to provide essential services, thereby securing the planet’s variety of life and contributing to human well-being and poverty eradication' (CBD 2010). Additionally, the fair and sustainable use of environmental resources, including genetic resources, to benefit all people, is paramount to the CBD framework (CBD 2010). To achieve Aichi targets, the UK has implemented the UK Post-2010 Biodiversity Framework published in 2012. The framework and its implementation plan set out 23 actions, each associated with one of the strategic goals of the Aichi targets. The framework also takes into account the EU Biodiversity Strategy.

In addition to the publication of Strategic Biodiversity Plans, the CBD COP has also developed seven thematic programmes of work, each focussing on a specific biome. Each programme of work highlights key issues and work needed within the field. The thematic programme of work on marine and coastal biodiversity covers four key elements, each divided up into operational objectives and implementation relies on each party much like the Strategic Plan for Biodiversity. Therefore, parties should set up relevant national and regional strategies and plans to enact the programme of work (CBD 2016).

The CBD has also adopted relevant designations such as ecologically or biologically significant areas (EBSA) to increase the attention brought to significant marine habitats. This term, adopted during COP 9 in 2008, is defined by nine scientific criteria: uniqueness or rarity, special importance for life history stages of species, importance for threatened, endangered or declining species and/or habitats, vulnerability, fragility, sensitivity or slow recovery, biological productivity, and biological diversity and naturalness. States and governmental organisations are responsible for designating EBSAs (CBD Secretariat 2012). EBSA can be associated with the Vulnerable Marine Ecosystem (VME) designation, defined by the UN General Assembly resolution 61/105. The resolution calls on states and regional fishery management organisations to restrict destructive fishing practices by closing off areas where VMEs occur or might occur and require vessels to move away from an area when VMEs were suddenly found.

### Oslo-Paris Convention (OSPAR Convention)

The OSPAR Commission and Convention were initially formed in Oslo in 1972 as the Oslo Convention against waste dumping in the North-East Atlantic. It was subsequently broadened in 1974 during the Paris Convention to cover land-based sources and the offshore industry. The two conventions were then unified and updated in the 1992 OSPAR Convention and finally in 1998, a new annex was adopted to cover nonpolluting human activities that can adversely affect the sea. The OSPAR Convention therefore emphasises the effect of anthropogenic activities on marine ecosystems in the North-East Atlantic. The OSPAR Commission includes 16 contracting parties (including the UK) and is supported by official observers and a secretariat. OSPAR observers comprise environmental protection nongovernmental organisations, as well as industry and trade organisations.

The OSPAR Commission published the North-East Atlantic Environment Strategy 2010–2020 (NEAE Strategy), which highlights the need for an ecosystem approach implementation; specifically, activities under the NEAE Strategy should integrate the management of anthropogenic activities and be based on the best available scientific knowledge. Additionally, the NEAE Strategy lays out five thematic strategies to address the main threats identified to marine ecosystems in the North-East Atlantic: biodiversity and ecosystem, eutrophication, hazardous substances, offshore industry, and radioactive substances. Offshore oil and gas activities are of special interest within the NEAE Strategy. Specific objectives set to the offshore oil and gas industry include (1) the reduction of the volume of oil in produced water discharged to the environment by 2020, (2) the limitation of offshore chemical discharge and the substitution of chemicals to safer options when feasible and (3) the development of activities to safely and permanently store CO_2_ in appropriate geological formation (CO_2_ capture and storage). UK implementation of the NEAE strategies is coordinated by the Department for Environment, Food and Rural Affairs (JNCC 2016).

Table 1 provides a summary of international conventions relevant to the sustainable management of marine species and habitats within the UK, including a list of key objectives. Please refer to Fig. 1 for a summary schematic.

## Comparative case study

Due to the aforementioned complexity that exists within governance of UK marine environments, conservation-relevant directives, managing authorities and designations vary considerably according to site location and features present therein. For illustration, we provide a simple comparison of two marine features of high conservation value (e.g. PMFs in Scotland), which vary in their UK distribution (including depth): deep-sea sponge grounds (found in offshore Scotland) and *Modiolus modiolus* (horse mussel) reefs (found across the UK). Consequently, these features face widely varying governance requirements as outlined in Table 2.

**Table 2:** Comparison of UK marine governance for meeting conservation objectives of two marine features of high conservation value in UK waters: deep-sea sponge grounds and coastal *Modiolus modiolus* (horse mussel) reefs

Feature	Deep-sea sponge grounds	*Modiolus modiolus* (horse mussel) reefs
Distributions	Faroe–Shetland Channel, Rosemary Bank, Hatton Bank and Rockall Basin (Scotland)	Scotland, Wales, Northern Ireland (various sites)
Coastal/offshore	Offshore	Coastal
Depth range	400–1400 m	0–49 m (commonly, though have been documented at greater depths)
Protected feature	Deep-sea sponge grounds composed of boreal osturs, bird nest sponge aggregations or stalked sponge aggregations	Annex I habitat: Reefs (Habitats Directive)
Key designations	Offshore NCMPA (Scotland), EC Natura 2000 SAC	EC Natura 2000 SAC, NCMPA (Scotland), MNR (Northern Ireland)
Other designations	Scottish Priority Marine Feature, Ecologically and Biologically Significant Area, Vulnerable Marine Ecosystem	OSPAR Priority Habitat (threatened/declining), Scottish Priority Marine Feature, UK Priority Habitat (previously UKBAP)
Key legislative directives	UK Marine and Coastal Access Act (2009) The Offshore Marine Conservation (Natural Habitats &c.) Regulations 2007	Habitats Directive (92/43/EEC) UK Marine and Coastal Access Act (2009) The Conservation (Natural Habitats, &c.) Regulations (1994) Marine (Scotland) Act 2010 Marine Strategy Framework Directive (2008/56/EC) The Marine Act (Northern Ireland) 2013
Key governing authorities	EC, DEFRA, JNCC, Marine Scotland	EC, DEFRA, JNCC, Marine Scotland, Northern Ireland Environment Agency, Welsh Government

### Summary

Marine conservation and management in the UK are driven by governance occurring at multiple levels, including within each of the four countries constituting the UK, over the UK as a single entity, via membership in the EU and according to international environmental agreements. Due to overlap between regional, national and international directives and agreements, it can be difficult to fully recognise the specific governance that may apply to a specific area or feature. Furthermore, the forthcoming implications of Brexit, including unknown consequences to policies derived from EU directives, may add additional complexity to determining how sites/features should be considered from a conservation management point of view. While Brexit will undoubtedly lead to changes in management plans in the UK, the severity of these changes remains to be seen. Regardless, improved understanding of current marine governance across the UK will aid in ensuring resources are managed in an economically and environmentally sustainable manner and help to safeguard features and habitats of high conservation value. Furthermore, clarification of existing management frameworks will aid transition to effective marine management under a post-Brexit UK and thereby support future sustainable marine developments across the UK.
